# Improved Estimation of Human Lipoprotein Kinetics with Mixed Effects Models

**DOI:** 10.1371/journal.pone.0138538

**Published:** 2015-09-30

**Authors:** Martin Berglund, Martin Adiels, Marja-Riitta Taskinen, Jan Borén, Bernt Wennberg

**Affiliations:** 1 Department of Mathematical Sciences, Chalmers University of Technology and the University of Gothenburg, Göteborg, Sweden; 2 Department of Molecular and Clinical Medicine, University of Gothenburg, Göteborg, Sweden; 3 Department of Medicine, Cardiovascular Research Unit, Diabetes and Obesity Research Program, Heart and Lung Center, University of Helsinki, Helsinki, Finland; Katholieke Universiteit Leuven, BELGIUM

## Abstract

**Context:**

Mathematical models may help the analysis of biological systems by providing estimates of otherwise un-measurable quantities such as concentrations and fluxes. The variability in such systems makes it difficult to translate individual characteristics to group behavior. Mixed effects models offer a tool to simultaneously assess individual and population behavior from experimental data. Lipoproteins and plasma lipids are key mediators for cardiovascular disease in metabolic disorders such as diabetes mellitus type 2. By the use of mathematical models and tracer experiments fluxes and production rates of lipoproteins may be estimated.

**Results:**

We developed a mixed effects model to study lipoprotein kinetics in a data set of 15 healthy individuals and 15 patients with type 2 diabetes. We compare the traditional and the mixed effects approach in terms of group estimates at various sample and data set sizes.

**Conclusion:**

We conclude that the mixed effects approach provided better estimates using the full data set as well as with both sparse and truncated data sets. Sample size estimates showed that to compare lipoprotein secretion the mixed effects approach needed almost half the sample size as the traditional method.

## Introduction

Complex traits (e.g. metabolic pathways and fluxes) may often only be quantified indirectly using mathematical or statistical models. A common approach is to make a parameterized mathematical model describing the system on an individual level, and then use statistical methods to make inference from the estimated parameters on the population level. Thus, any uncertainty in the individual estimate is affecting the population estimate. The analysis is further complicated by the great variability seen in biological systems.

There are at least two conceptually different approaches to treat variability in mathematical models of biological systems. In the *standard two stage* (STS) approach [1,2,3], each individual is treated separately: measured data for an individual are used to estimate parameters that are assumed to be truly specific for that individual (the first stage). The obtained individual parameters are then treated with traditional statistical methods (the second stage), such as correlations with other covariates or comparison of parameters between groups.

Contrary to this, in a *mixed effects model* one assumes that each parameter in the model is either essentially the same for all individuals in the group, a function of some directly quantified individual-specific traits (covariates), or random with a probability distribution that is determined by covariates and parameters that are common for the whole group. One can then use all measured data to make parameter estimations for the groups.

An advantage of the STS approach is that it does not assume any *a-priori* information on the distribution of the parameters and on treatment effects (i.e. which model parameters are affected by treatment) or group effects (i.e. which model parameters are different between groups). A disadvantage is that, to adequately estimate parameters for each individual, large data sets are needed and to estimate treatment or group differences, large sets of individuals are needed. In case of complex and costly experiments, a modeling approach that utilizes smaller data sets is desirable to reach sufficient study power while minimizing the cost of the experiment.

The mixed effects, including nonlinear mixed effects (NLME), approach is widely described and used in the literature [3,4,5,6,7,8]. It has been shown that better estimates of population characteristics are obtained for mixed effects models compared to STS models in many systems. Sheiner and Beal showed already in 1980 that the STS method can produce biased and imprecise estimates of the variability between individuals [4]. The STS and mixed effects approaches have been compared for many systems [2,9,10,11].

Abnormal concentrations of blood lipids and lipoproteins are key risk factors for cardiovascular disease and are often observed in metabolic diseases such as diabetes mellitus type 2 (DM2) [12]. In DM2, the lipid abnormalities primarily include elevated plasma triglycerides and decreased high density lipoprotein (HDL) cholesterol [12] and it is believed that these changes are the cause of increased risk in cardiovascular disease in DM2 and related diseases [12,13]. Many therapies are targeted to normalize blood lipid levels, and potentially decrease the risk for developing cardiovascular disease.

The non-soluble lipids, such as cholesterol esters and triglycerides, are transported in the core of specific vesicles called lipoproteins. On the surface of these particles different proteins are attached. During fasting, these particles are synthesized in the liver and transport the lipids to peripheral tissues. As the lipids are delivered the particles become smaller and denser as the relative amount of protein (which is denser than the lipids) increases. The catabolic processes are combinations of lipases (lipoprotein lipase and hepatic lipase), which remove triglycerides from the core, and removal of whole particles due to specific uptake mechanisms. Thus, elevation of plasma triglyceride in disease states may be attributed to increased secretion, defined by the secretion rate (SR), or increased clearance.

To understand disease development and the role between the underlying metabolic defects and blood lipids levels the dynamics of lipid and lipoproteins are studied using stable isotope infusions and mathematical modeling [14]. Using such models, quantification of both the catabolic processes and the productions is possible [14].

Compartmental models are widely used to describe the synthesis, assembly and kinetics of lipoproteins [14,15,16,17,18,19,20,21,22,23]. Time series data are generated by infusion of tracers, typically either labeled glycerol [17,20,21,22], amino acids [18,19,23], or combinations of both [15]. The amounts of the labeled and unlabeled tracers are then measured by gas chromatography / mass spectroscopy (GC/MS).

The models allow for the quantification of the secretion rate (SR) of lipoproteins (either protein or triglyceride content) (mg/kg bw/day), the fractional clearance rate (FCR) representing the total turnover of the lipoproteins (pools/day), the fractional transfer rate (FTR) representing the fractional conversion of large particles to smaller particles due to the action of lipases (pools/day) and the fractional direct catabolic rate (FDCR) representing the direct catabolism/removal of large particles (pools/day).

Data acquisition is labor intensive, in particular when the system is described by several sub-fractions. Improved methods for data analysis may therefore allow for a reduction of either the number of study subjects or the amount of data needed for each subject while maintaining study power.

In this paper we compare the traditional STS approach and a new mixed effects model approach. We use experimental data from fifteen healthy control subjects and fifteen DM2 subjects. DM2 is typically associated with elevated plasma triglyceride, which is known to be caused by increased production of lipoprotein particles from the liver [24,25,26,27]. The aim of the study was to investigate if the mixed effects approach improved parameter estimation compared to the STS approach for small sample sizes and for sparse data. We base the analysis on the established model of lipoprotein kinetics [15,19,23].

We show that, when shrinking the sample size, group properties are preserved using the mixed effects approach while the STS model fails to replicate significant differences between the groups. Moreover, individual estimates are better when using the mixed effects approach while using less data for each individual.

## Results

### The Model

**Fig 1:**
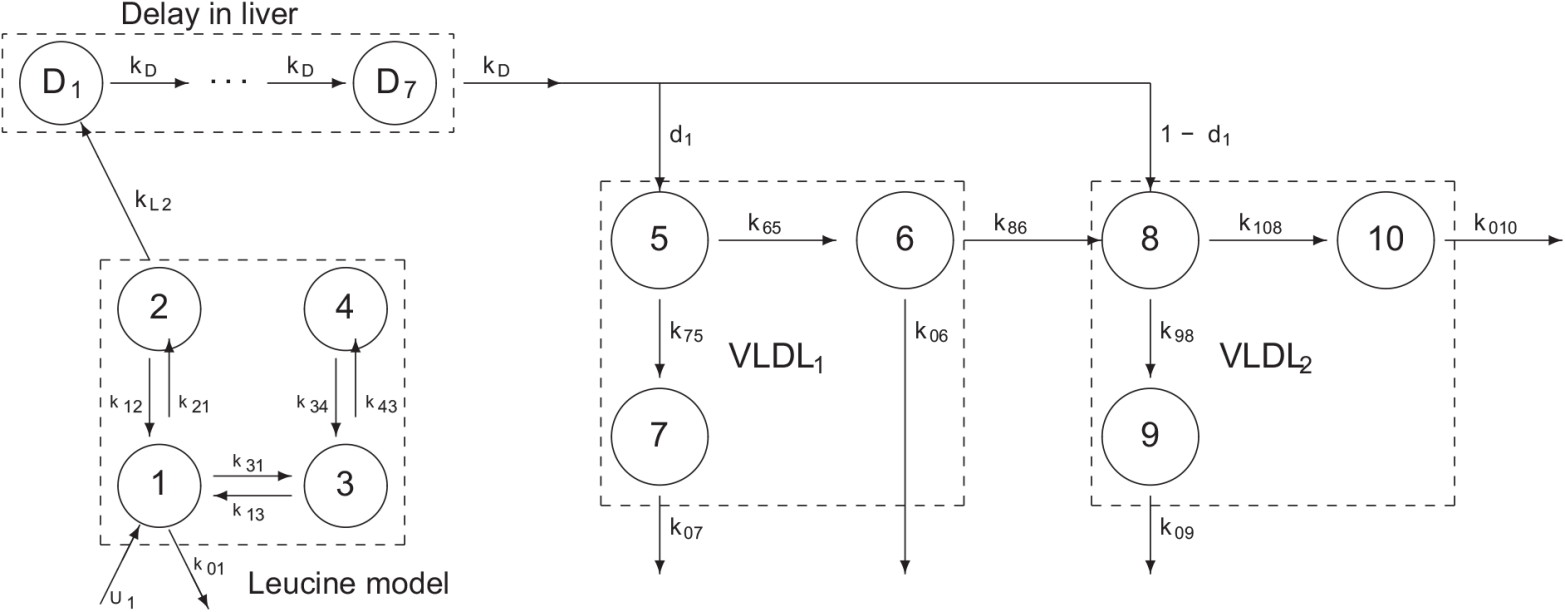
Model structure of compartmental model. The same structural model was used for all individuals and in both the mixed effects and the STS model. Model constraints, *k*_2,1_ = *k*_1,2_, *k*_3,4_ = 0.1 *k*_4,3_, *k*_9,8_ = *k*_7,5_ and *k*_0,9_ = *k*_0,7_ were used. Compartment 1 represents the free leucine in the plasma, compartments 3 and 4 represents leucine recycling in non-hepatic tissue and compartment 2 is the intrahepatic leucine that feeds the apoB synthesis compartment represented as a delay (D_1_-D_7_). Newly synthesized apoB enters the plasma as VLDL_1_ (large particles, compartment 5) or VLDL_2_ (small particles, compartment 8). VLDL_2_ may also be produced through conversion of VLDL_1_ via compartment 6. Particles may leave the system through compartments 6, 7, 9 or 10.

In this paper we develop a mixed effects population model of the very low density lipoprotein subclass (VLDL_1_ and VLDL_2_) kinetics. The model is based on our previous work on individual compartmental models [15], see **Fig 1**. The details of the model are described in the *Materials and Methods* section.

We distinguish between *model parameters* (e.g. transfer rates in the model) and *key parameters* (e.g. biologically relevant parameters, often composed of several model parameters).

The data set consists of 15 healthy control subjects and 15 patients with DM2, as characterized in **S1 Table**.

### Estimated Parameters Using All Subjects

Three data sets are used. The full data set (all data) and two reduced data sets: the first 4 hours (*truncated data*) and every second data point of the VLDL tracer data (*reduced data*).

The estimated key parameters using both the STS and the mixed effects approaches and the three data sets are summarized in **Table 1**.

**Table 1:** Estimations of key parameters using either the STS or the mixed effects modeling approaches.

DATA	METHOD	GROUP	VLDL_1_ FCR	VLDL_1_ FTR	VLDL_1_ FDCR	VLDL_1_ SR	VLDL_1_ pool	VLDL_2_ IndSR	VLDL_2_ FCR	VLDL_2_ DSR	VLDL_2_ pool	Delay
**All**												
	STS	Control	12.68 (51)	5.63 (44)	6.05 (101)	8.7 (44)	0.69 (58)	3.86 (54)	5.31 (41)	2.33 (34)	1.21 (49)	0.5 (27)
	STS	DM2	9 (55)	4.6 (53)	4 (82)	13.13 (36)**	1.46 (57)***	6.71 (42)**	4.85 (48)	2.38 (58)	1.93 (42)**	0.42 (16)*
	NLME	Control	13.54 (46)	4.82 (33)	7.18 (115)	9.3 (26)	0.69 (56)	3.31 (75)	5.04 (23)	2.51 (24)	1.21 (50)	0.47 (22)
	NLME	DM2	10.95 (41)	6.27 (40)*	4.27 (69)*	16.08 (28)***	1.47 (54)***	9.21 (24)***	6.49 (32)*	2.91 (49)	1.92 (43)**	0.38 (21)*
**Truncated**												
	STS	Control	15.51 (50)	7.04 (49)	7.01 (100)	11.27 (40)†	0.73 (64)	5.12 (68)	6.94 (44)†	2.81 (34)	1.18 (50)	0.49 (29)
	STS	DM2	11.15 (49)	5.17 (76)	5.16 (89)	16.42 (52)*	1.47 (57) **	7.61 (47)	5.89 (45)	2.98 (63)	1.92 (45)**	0.41 (22)
	NLME	Control	13.94 (48)	3.72 (38)†	9.05 (87)	10.11 (27)	0.73 (60)	2.7 (84)	4.7 (17)	2.56 (23)	1.18 (52)	0.47 (24)
	NLME	DM2	11.6 (49)	4.55 (65)†	5.98 (86)	17.03 (29)***	1.47 (55)**	6.69 (39)***,‡	5.26 (38)	2.94 (49)	1.93 (46)**	0.36 (19)**
**Reduced**												
	STS	Control	14.49 (66)	4.58 (55)	8.27 (117)	9.95 (41)	0.69 (58)	3.14 (82)	4.92 (37)	2.3 (38)	1.21 (49)	0.5 (22)
	STS	DM2	10.5 (63)	4.5 (37)	5.39 (107)	15.28 (52)*	1.46 (57)***	6.55 (49)**	4.99 (47)	2.58 (88)	1.93 (42)**	0.43 (24)
	NLME	Control	15.12 (51)	5.03 (21)	8.81 (103)	10.37 (22)	0.69 (56)	3.45 (62)	5.08 (20)	2.5 (21)	1.21 (49)	0.5 (17)
	NLME	DM2	11.39 (40)	6.11 (35)	4.85 (70)	16.67 (27)***	1.46 (53)***	8.94 (27)***	6.36 (30)*	2.94 (53)	1.92 (44)**	0.4 (21)**

Healthy control subjects (Control) and type 2 diabetic patients (DM2) are compared using the two approaches and using three sets of data: The complete data set (All), the truncated data set (up to 4 hours, Truncated), and the reduced data set (every second data point removed, Reduced). The estimated parameters are presented as geometric mean (coefficient of variation). Differences between Control and DM2 were compared using t-test after log-transforming the data. FCR, fractional catabolic rate; FTR, fractional transfer rate; FDCR, fractional direct catabolic rate; SR, secretion rate; indSR, in-direct secretion rate (ie flux from VLDL_1_), DSR, direct secretion rate.

*, p<0.05;

**, p<0.01;

***, p<0.001 (versus Control within same model and data set).

†, p<0.05,

‡, p<0.01 (versus All data within the same group and model).

#### All data

Using the full dataset the mixed effects approach typically produced smaller variations within groups compared with the STS approach (**Table 1**). Both approaches confirmed previous findings of greater secretion of VLDL_1_ particles (VLDL_1_ SR), resulting in a larger VLDL_1_ pool, a greater flux from VLDL_1_ to VLDL_2_ (expressed as indirect secretion of VLDL_2_), and a larger VLDL_2_ pool in the DM2 patients compared with the control subjects (**Table 1**). With both approaches the total VLDL_1_ FCR was lower in DM2 patients compared with control subjects. However, the difference was not significant. In contrast to the STS approach, the mixed effects model showed significantly higher VLDL_1_ FTR and lower VLDL_1_ FDCR in DM2 patients compared with the control subjects (**Table 1**).

**Fig 2:**
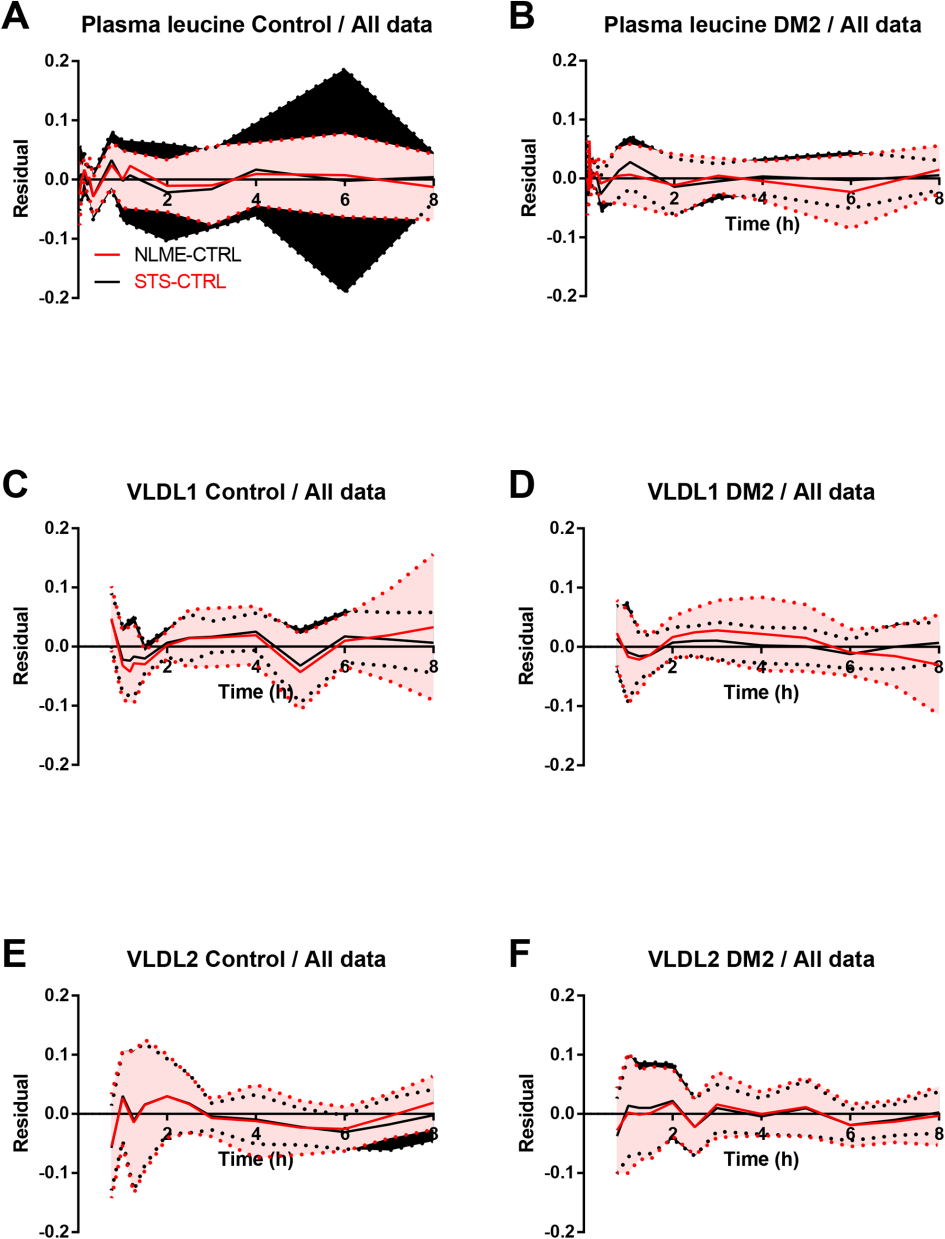
Residual plots of enrichment data using all data. The residuals (model fit minus measurement data) for the three enrichment data sets (A and B, plasma leucine; C and D, VLDL_1_; E and F VLDL_2_) were plotted for the two methods (STS and NLME) and the two groups (A, C and E, Control; B, D and F, type 2 diabetes mellitus (DM2)). Both methods produced good fits to the data. The NLME approach used a sequential procedure; the plasma leucine were fitted first and the VLDL_1_ and VLDL_2_ curves were fitted using the leucine results. This may explain the worse fit for the plasma leucine in the STS approach. Lines, mean of mixed effects approach (red) and STS approach (black); Areas, mean ± SD for mixed effects approach (red) and STS approach (black).

Both approaches produced excellent fit to the data as seen in the residual plots (**Fig 2A–2F**). The STS approach generally produced slightly better fit to the data when quantified using the root mean square error (RMSE) of the residuals for all individuals.

#### Truncated data

The mixed effects model replicated all significant differences between the DM2 patients and the control subjects compared with using the full data set (**Table 1**). Using the truncated data, the STS model still showed higher VLDL_1_ SR in DM2 patients although with lower significance, but did not detect a higher VLDL_1_ to VLDL_2_ transfer for DM2 patients compared with control subjects (Table 1). In general the mixed effects model generated data with smaller CV (**Table 1**), indicating a better coherence between the results using the mixed effects approach.

Comparing the mixed effects model using all data with the truncated data showed significantly lower VLDL_1_ FTR in both control subjects (p<0.05) and in DM2 patients (p<0.05) using truncated data. In addition, the VLDL_1_ to VLDL_2_ transfer estimates were lower with truncated data in DM2 patients (p<0.01) (**Table 1**).

Using truncated data, the STS estimates of VLDL_1_ SR were significantly higher (p<0.05) and the VLDL2 FCR were significantly lower (p<0.05) in control subjects, compared with using all data (**Table 1**).

The mixed effects model produced better fit to the data even when extrapolating the model curves to the excluded data (S1A–S1F Fig). This indicates that, in at least some individuals, the information about the trajectories is captured by the information in the first 4 hour data and that the mixed effects approach shares this information to provide better fit for all individuals.

#### Reduced data

After reduction of data by removing every second data point both methods still reproduced the major findings of study, however with higher significance for the mixed effects model compared with the STS model (**Table 1**). Using the reduced data sets no estimate were significantly different using neither the mixed effects model nor the STS model (**Table 1**). The fit to the curves were similar for the two methods (**S2A–S2F Fig**).

### Reduced Study Sample Size

As the mixed effects model produced smaller variations we next tested how this affected the sample size needed to detect a difference between the groups. The (log-transformed) means and variances estimated using the full data set were used to calculate the sample size needed to detect a difference between the means with a power of 80%. To further test this we also used a resampling approach to resample the study population into smaller group sizes of 4, 6, 8, 10, and 12 subjects from each group. Both the mixed effects model and the STS model were applied to the re-sampled subgroups and the p-values for the comparison between the control subjects and the DM2 patients were recorded for 30 different re-sampled groups for each group size.

The estimated sample sizes were generally smaller for the mixed effects approach compared with the STS approach, both using the sample size calculation and the resampling approach (**Table 2**).

**Table 2:** Sample size analysis.

	Parameter	VLDL_1_ FCR	VLDL_1_ FTR	VLDL_1_ FDCR	VLDL_1_ SR	VLDL_1_ pool	VLDL_2_ IndSR	VLDL_2_ FCR	VLDL_2_ DSR	VLDL_2_ pool	Delay
**NLME**											
	p-value using all data	0.178	0.0498	0.0804	<0.0001	0.0004	0	0.0171	0.2819	0.0081	0.0279
	Estimated sample size	33	17	20	5	6	5	12	51	10	10
	Estimate of sample size (resampled)	NA	NA	NA	6	8	8	NA	NA	12	NA
**STS**											
	p-value using all data	0.0686	0.2346	0.1566	0.0068	0.0006	0.0027	0.573	0.8952	0.0065	0.0425
	Estimated sample size	19	42	30	9	7	8	183	3334	9	16
	Estimate of sample size (resampled)	NA	NA	NA	10	8	10	NA	NA	12	NA

P-values for the difference between the groups were first calculated using all data, significant differences and the means and variances were considered as true. Desired sample sizes with study power of 80% were first estimated directly, using the estimated means and variances. The comparisons between healthy control subjects and DM2 patients were also repeated with both the mixed effects model and the STS model in re-sampled groups of 4, 6, 8, 10 and 12 subjects. For each group size the analysis was repeated 30 times. The minimum number of individuals needed to correctly identify a true difference at least 80% was considered as the minimal sample size. FCR, fractional catabolic rate; FTR, fractional transfer rate; FDCR, fractional direct catabolic rate; SR, secretion rate; indSR, in-direct secretion rate (ie flux from VLDL_1_), DSR, direct secretion rate.

Using the mixed effects approach the increased VLDL_1_ secretion in DM2 patients were correctly identified in all 30 resampled iterations using the mixed effects model for a study group size of 12 and 10. The difference was correctly identified in more than 80% of the iterations down to a group size of 6 subjects. In contrast the STS approach reached an 80% correct identification rate at a group size of 10. This indicates that to have an estimated study power of 80% in detection of the difference in VLDL_1_ SR it is sufficient to have only 6 subjects from each group using the mixed effects approach but 10 using the STS approach (**Table 2**). Similar results were achieved for the VLDL_1_ to VLDL_2_ transfer and VLDL_1_ pool (**Table 2**). The sample size was also estimated directly using power calculations (**Table 2**) with comparable results.

### Computational Power

A major concern with mixed effects modeling is the extended computational time needed to estimate the likelihood function. For each calculation of the likelihood function, the individual parameters need to be estimated. Some steps can therefore be parallelized. Using our implementation in Matlab, a mixed effects model with 8 DM2 and 8 control subjects could still not be run on a traditional PC. On an 8 core cluster the computational time was between 24h and 42h in the 30 repeated sample size estimates, compared to less than 10 minutes for the STS model.

## Discussion

The main result in this study is the novel application of mixed effects models to experimental data from human lipoprotein kinetic studies, comparing the lipid metabolism in healthy control subjects and DM2 patients. Compared to the traditional STS approach our mixed effects approach provided stronger results using the full data sets. The results also provide a basis for the use of smaller sample sizes when data are analyzed with the mixed effects approach.

The mixed effects approach produced estimates with less variation which translated into the possibility to use fewer subjects with sustained study power as shown by direct calculations as well as simulations using resampled populations. Furthermore we show that significant results may be obtained using smaller experimental datasets, as given either by either shorter sampling times or less frequent sampling.

Studies of lipoprotein kinetics are labor intensive and require generation of large sets of data for each individual in order to obtain good parameter estimates. Often subjects are studied for several different tracers simultaneously which adds to the complexity. It is therefore of great interest to derive novel techniques that enables better estimates of kinetic parameters.

Many metabolic diseases are linked to disturbances in lipid metabolism. The traditional analysis of plasma concentrations cannot answer which underlying mechanism that is responsible for the observed disturbance [14]. In contrast lipoprotein kinetic studies allows for decomposition of the concentration into several components including secretion and clearance. In the case of type 2 diabetes, there is a large body of evidence that the major defect resulting in elevated plasma triglycerides is an increased secretion of triglyceride rich lipoproteins ([24,25,26,27,28]). The rate of clearance, however, is usually lower in type 2 diabetic patients but the results are inconsistent with studies showing either non-significant (although with a trend) [24,26,28] or significant [25] differences. Our results confirm these results and may further contribute the analysis by providing better estimates of variability of the clearance parameters.

We tested the effectiveness of the mixed effects model using two different approaches to reduce data. First we reduced the data by either truncating the time series or by removing every second data point. Truncating the data results in loss of information about the tail shape of the curves, which usually lead to poorer estimates. The mixed effects model provided better predictions of the excluded data on an individual basis. Both the mixed effects approach and the STS approach showed significant differences in the estimated means compared to the full data sets (Table 1) for some parameters indicating that the absolute quantifications were poorer using the truncated data. However both approaches consistently recreated the significant differences between the DM2 patients and the control subjects as observed with all data, with p-values being smaller using the mixed effects approach. When data were instead reduced by 50% by removing every second data point, the absolute quantifications were not different compared with those obtained using all data. The mixed effects approach provided smaller p-values for the significant differences between the two groups.

The second approach to compare the two methods was to perform a power calculation and to estimate the sample size needed to detect differences with a power of 80%. The mixed effects approach needed smaller sample size compared with the STS approach, both when the sample size were calculated directly or when estimated using resampling.

An interesting observation was that when truncating the data at 4 hours, the mixed effects approach predicted the measurements between 4 and 8 hour much better than the STS approach. Typically subjects with normal plasma triglycerides does not present a “tail” of enrichment in VLDL_1_ but subjects high plasma triglycerides do [19]. To account for these slowly turning over lipoproteins the compartments 7 and 10 are added to the model. It appears as the mixed effects model may capture this behavior in some individuals already before 4 hours and share this among all individuals.

This is consistent with the fact that the STS approach estimates both the VLDL_1_ SR and VLDL_1_ FCR higher when truncated data is used, suggesting an inability to capture a slowly turning over pool.

We tested the mixed effects approach on an observational study. For interventional studies the statistical models for the parameters in the population model needs to be defined according to the study design.

Our mixed effects model was implemented in MATLAB. The computational time for the mixed effects approach is several magnitudes slower compared to the STS approach. This may be attributed to the fact that in each calculation of the maximum likelihood function individual parameters needs to be estimated for each individual. It is therefore not today feasible to run this on a traditional workstation PC. However as the estimation of the individual parameters are independent, the calculations may be done in parallel threads and may therefore be optimized for cluster or multiple processor workstations. However, the additional costs in computation are likely minor compared to the possible savings in study population size, study power and/or laboratory work.

In conclusion, we shown that mixed effects modeling is an effective tool to estimate kinetic parameters in human lipoprotein kinetic studies. Either sample size or the amount of data may be greatly reduced while retaining study power.

## Materials and Methods

### Study Population

Data used in this study are previously published [15,24,29]. In this study, we randomly selected 15 healthy control subjects and 15 patients with type 2 diabetes with similar age and BMI (**S1 Table**) from our study cohorts. The diagnosis of DM2 was based on glucose tolerance test results in the diabetic range according to World Health Organization (1999) criteria or on the use of oral diabetes medication. The original study designs were approved by the Helsinki University Central Hospital ethics committee, and each subject gave written, informed consent as described in the original publications [15,24,29]. All samples were collected in accordance with the Helsinki Declaration.

### Data

The details of the experimental protocol, sample analysis etcetera are explained elsewhere [15,24]. In short, *N*_*C*_ = 15 healthy control subjects and *N*_*D*_ = 15 patients with type 2 diabetes with similar age and BMI were studied (**S1 Table**). Following an overnight fast a bolus infusion of [^2^H_3_]-leucine was infused. Blood was collected and the amounts of labeled and unlabeled leucine were measured as free in plasma and in the major protein component, apolipoprotein B-100 (apoB), in VLDL_1_ and VLDL_2_ by GC/MS. The tracer-to-tracee ratio (i.e. labeled-to-unlabeled leucine) was calculated for free leucine in plasma (sampled at 2, 4, 6, 8, 10, 12, 15, 20, 30, and 45 minutes and 1, 2, 3, 4, 6, and 8 hours after the injection) and as leucine in VLDL_1_ and VLDL_2_ (sampled at 0.5, 0.75, 1, 1.25, 1.5, 2, 2.5, 3, 4, 5, 6, 7, and 8 hours). Pool sizes of VLDL_1_ and VLDL_2_ are also measured at 0, 4, and 8 hours after the injection, and recalculated to leucine levels in apoB in VLDL_1_ and VLDL_2_ as explained in [15].

Three data sets are used. The full data set and two reduced data sets: the first 4 hours (*truncated data*) and every second data point (*reduced data*). Furthermore reduced group sizes (i.e. 12, 10, 8, 6 and 4 subjects) are tested.

The full data set is available as an online supplement in file **S1 File Data.csv** with data format given in **S2 File Data Format.docx**.

### The Individual Model

The individual model is based on the compartmental model formulation developed by Demant and Packard [19,23]. The model has been used to determine the kinetics of apoB-100 in the VLDL_1_ and VLDL_2_ subclasses in individuals with a wide range of conditions (e.g. [15,18,19,23,24,29]). The model has a 4 compartment catenary chain that models the free leucine in plasma and the intra-hepatic leucine pool before entering the synthesis module consisting of a seven compartment delay. Each sub-system of VLDL_1_ and VLDL_2_ have three compartments (5, 6 and 7 for VLDL_1_ and 8, 9 and 10 for VLDL_2_, **Fig 1**), two compartments are representing a delipidation chain (5 and 6, and 8 and 10 respectively) resulting in transfer to denser particles and one compartment (7 and 9 respectively) represents particles being cleared from the circulation by other mechanisms as summarized in **Fig 1**.

We denote the mass of the tracer (labeled leucine) for an individual *i*, in a compartment *j*, at time *t* by $q_{j}^{i}(t)$ and the constant mass of tracee (unlabeled leucine) by $Q_{j}^{i}$. Thus, free leucine is denoted by $q_{1}^{i}(t)$ and $Q_{1}^{i}$, VLDL_1_ leucine in apoB is denoted by $q_{V_{1}}^{i}(t)=q_{5}^{i}(t)+q_{6}^{i}(t)+q_{7}^{i}(t)$ and $Q_{V_{1}}^{i}(t)=Q_{5}^{i}(t)+Q_{6}^{i}(t)+Q_{7}^{i}(t)$, and VLDL_2_ leucine in apoB is denoted by $q_{V_{2}}^{i}(t)=q_{8}^{i}(t)+q_{9}^{i}(t)+q_{10}^{i}(t)$ and $Q_{V_{2}}^{i}(t)=Q_{8}^{i}(t)+Q_{9}^{i}(t)+Q_{10}^{i}(t)$.

To have comparable results we use the same model of error and method of parameter estimation for both the mixed effects model (below) and the individual model. The output variables are assumed to be log-normally distributed as this result in relative errors. Therefore the data are log transformed before analysis and the output variables for each sample time point *t*_*k*_ are defined by the logarithm of the corresponding variables with the added normally distributed error term as:

y1i(tk)=log(q1i(tk)q1i(tk)+Q1i)+e1i(tk),e1i(tk)~N(0,[sleui]2)yV1i(tk)=log(qV1i(tk)qV1i(tk)+QV1i)+eV1i(tk),eV1i(tk)~N(0,[sV1i(tk)]2)yV2i(tk)=log(qV2i(tk)qV2i(tk)+QV2i)+eV2i(tk),eV2i(tk)~N(0,[sV2i(tk)]2)YV1i(tk)=log(QV1i)+EV1i(tk),EV1i(tk)~N(0,[SV1i]2)YV2i(tk)=log(QV2i)+EV2i(tk),EV2i(tk)~N(0,[SV2i]2)

We assume that the errors are of the same magnitude for VLDL_1_ and VLDL_2_, i.e. $s_{V_{1}}^{i}(t_{k})=s_{V_{2}}^{i}(t_{k})≔s^{i}(t_{k})$ and $S_{V_{1}}^{i}(t_{k})=S_{V_{2}}^{i}(t_{k})≔S^{i}(t_{k})$. Since only a small amount of tracer material have been incorporated into the lipoproteins at 0.5 hours (the time point for the first measurement of tracer kinetics of VLDL_1_ and VLDL_2_), the uncertainty is larger for measurements taken after only half an hour. Therefore, we assume that

$$s^{i}(t_{k})=\{\begin{array}{cc} \phi^{i}s^{i} & t_{k}=0.5\\ s^{i} & t_{k}\neq 0.5 \end{array}$$

for a parameter *ϕ* ^*i*^ ≥ 1. In principle, separate values of *s* ^*i*^(*t*_*k*_) could be used for each *k*. The state variables are given by solving the following system

$$\begin{array}{c} \frac{dq^{i}}{dt}=K^{i}q^{i}\\ \;0=K^{i}Q^{i}+U^{i} \end{array}.$$ (1)

where *q* ^*i*^ and *Q* ^*i*^ are state vectors for the tracer- and tracee system respectively, *U* ^*i*^ is the inflow vector of tracee material into the system (which is only through compartment 1 for this model), and *K* ^*i*^ is the compartmental matrix of the model (see, e.g., [30,31]). The steady-state assumption implies that the left hand side in Eq (1) is zero. The unknown model parameters to estimate for each individual are $k_{0,1}^{i},k_{1,2}^{i},k_{1,3}^{i},k_{3,1}^{i},k_{4,3}^{i},Q_{1}^{i},p_{1}^{i}$ (the fraction of leucine in the VLDL synthesis machinery that comes from free leucine in plasma), $k_{L,2}^{i},k_{D}^{i},d_{1}^{i},k_{6,5}^{i},k_{8,6}^{i},k_{7,5}^{i},k_{0,7}^{i},k_{10,8}^{i},k_{0,10}^{i},$and also $s_{leu}^{i},\phi^{i},s^{i},$ and *S* ^*i*^. $U_{1}^{i}$and $Q_{j}^{i},j\neq 1$ can be derived from Eq (1) if *K* ^*i*^ and $Q_{1}^{i}$ are known.

### The Population Model

For the STS approach each parameter in the model is estimated individually (except *ϕ* ^*i*^, which is set to 10). Key variables are then derived from the model parameters as is explained below. For the mixed effects approach the model parameters for all individuals are estimated simultaneously by assuming that the individual model parameters are members of probability distributions determined by the whole population. For this mixed effects model we separate the leucine model to the rest and first estimate the parameters in the leucine model as carefully explained in [32]. For the VLDL kinetics part of the model the individual model parameters are

$$\begin{array}{l} \;p_{1}^{i}=(1_{C}^{i}p_{1}^{C}+1_{D}^{i}p_{1}^{D})\exp(\eta_{p_{1}}^{i}),\\ \;k_{d}^{i}=(1_{C}^{i}p_{d}^{C}+1_{D}^{i}p_{d}^{D})\exp(\eta_{d}^{i}),\\ \;k_{6,5}^{i}=(1_{C}^{i}k_{6,5}^{C}+1_{D}^{i}k_{6,5}^{D})\exp(\eta_{6,5}^{i}),\\ \;k_{7,5}^{i}=(1_{C}^{i}k_{7,5}^{C}+1_{D}^{i}k_{7,5}^{D})\exp(\eta_{7,5}^{i}),\\ \;k_{0,6}^{i}=\eta_{0,6}^{i},\\ k_{0,10}^{i}=(1_{C}^{i}k_{0,10}^{C}+1_{D}^{i}k_{0,10}^{D})\exp(\eta_{0,10}^{i}),\\ \;k_{0,9}^{i}=k_{0,7}^{i},\\ \;k_{L,2}^{i}=(1_{C}^{i}k_{L,2}^{C}+1_{D}^{i}k_{L,2}^{D})\exp(\eta_{L,2}^{i}),\\ \;d_{1}^{i}=(1_{C}^{i}d_{1}^{C}+1_{D}^{i}d_{1}^{D})\exp(\eta_{d_{1}}^{i}),\\ \;k_{8,6}^{i}=(1_{C}^{i}k_{8,6}^{C}+1_{D}^{i}k_{8,6}^{D})\exp(\eta_{8,6}^{i}),\\ \;k_{0,7}^{i}=(1_{C}^{i}k_{0,7}^{C}+1_{D}^{i}k_{0,7}^{D})\exp(\eta_{0,7}^{i}),\\ k_{10,8}^{i}=1_{C}^{i}k_{0,10}^{C}+1_{D}^{i}k_{0,10}^{D},\\ \;k_{9,8}^{i}=k_{7,5}^{i},\\ \;s^{i}=s,\phi^{i}=\phi ,\;S^{i}=S, \end{array}$$ (2)

where each $\eta_{x}^{i}\sim N(0,\omega_{x}^{2})$, except $\eta_{0,6}^{i}$ which is allowed to vary uniformly between 0 and 5, since we could not obtain good fits to the data for all individuals with a normally distributed $\eta_{0,6}^{i}$. The indicator functions $1_{C}^{i}$ and $1_{D}^{i}$ are used to indicate if individual *i* belongs to the control- or DM2 group. Observe that we assume that all *ω*_*x*_ are group independent. The reason for using fixed population parameters for *k*_10,8_ is that this parameter is hard to estimate when only VLDL_1_ and VLDL_2_ data are available. Better fits to the data were not obtained for individual-specific *k*_10,8_ (for the STS models the parameters for each subject were re-estimated with *k*_10,8_ fixed to the group values obtained from the mixed effects model). In fact, *ω*_10,8_ tended to zero when it was included in the mixed effects model. The variability parameters in the residuals are assumed to be identical for all individuals, Eq (2). A final remark is that we assume that the covariance matrix Ω of $\eta^{i}=\left[\eta_{p_{1}}^{i},\ldots ,\eta_{0,10}^{i}\right]$ is diagonal to keep a reasonable dimension of the parameter space.

### Geometric Mean and Coefficient of Variation of a Log-Normal Distribution

Here we explain how the population values of the parameters in the tables and figures are computed. Let *θ* ^*i*^, *i* = 1,…,*N* be the estimated values for a parameter for the subjects in one of the groups. Almost all key parameters are skewed over the population (for both the STS- and mixed effects models). Therefore, we assume that they are log-normally distributed and compute the sample geometric mean

$${\left(\prod_{i=1}^{N}\theta^{i}\right)}^{\frac{1}{N}}$$ (3)

which is the maximum likelihood estimator for the median of a log-normal distribution [33]. The coefficient of variation (in percent) is given by

$$100\sqrt{\exp({\hat{\omega }}^{2})-1},$$

where ${\hat{\omega }}^{2}$ is the unbiased estimate of the sample variance of the logarithm of the *θ* ^*i*^‘s. When statistical tests are performed we compare logarithms of the parameters and use the conventional t-test theory. This can be done since the logarithm of the geometric mean in Eq (3) is $\frac{1}{N}\sum_{i=1}^{N}\log(\theta^{i})$.

### Parameter Estimation

For parameter estimation of the mixed effects model, we apply the maximum likelihood framework. Here we quickly present how the likelihood function of the population parameters is approximated. For a more thorough analysis and further references we refer to [32,34]. The population likelihood function is a marginal likelihood function, where the vector $\eta^{i}\in {ℜ}^{r}$ of all individual-specific parameters (*r* = 9 for the VLDL model) are integrated out. Let *θ* be the vector of population parameters and let *Y* = [*Y* ^1^,…,*Y* ^*N*^] the complete set of measurement data for the *N* individuals. Assuming that the subjects are picked independently in the population the population likelihood function takes the form

$$L(\theta ;Y)=\prod_{i=1}^{N}\int_{{ℜ}^{r}}\exp(l^{i})d\eta^{i},$$ (4)

where *l* ^*i*^ = *l* ^*i*^(*η* ^*i*^) = *l* ^*i*^(*η* ^*i*^;*Y* ^*i*^,*θ*) is the log-likelihood function for subject *i*. Let the residuals at time *t*_*k*_ be denoted $\varepsilon_{k}^{i}$ and let $R_{k}^{i}$ be its covariance matrix, hence $R_{k}^{i}=diag(s{\left(t_{k}\right)}^{2},s{\left(t_{k}\right)}^{2},S^{2},S^{2})$ for the VLDL model. The individual log-likelihood function is given by

$$l^{i}=-\frac{1}{2}(\sum_{k=1}^{d^{1}}\left[\varepsilon_{k}^{i}{}^{{}^{T}}R_{k}^{i}{}^{{}^{-1}}\varepsilon_{k}^{i}+\log\left|R_{k}^{i}\right|+m\log(2\pi )\right]+\eta^{i}{}^{{}^{T}}\Omega^{-1}\eta^{i}+\log\left|\Omega \right|+r\log(2\pi )),$$ (5)

where *m* is the number of outputs (thus, *m* = 1 for the free leucine model and *m* = 4 for the VLDL model). The integrand, exp(*l* ^*i*^), in Eq (4) is hard or impossible to integrate exactly and must therefore be approximated. The Laplace approximation method is based on a second order truncation of the Taylor expansion of *l* ^*i*^ around a stationary point ${\hat{\eta }}^{i}$ (see, e.g., [32,34] for the derivations). Eq (4) becomes

L(θ;Y)≈∏exp(li(η^i))|Hη(li(η^i))(2π)r|−12, (4b)

where $H_{\eta }(l^{i}({\hat{\eta }}^{i}))$ is the Hessian matrix of *l* ^*i*^ with respect to *η* ^*i*^. In the first order methods second order derivatives are disregarded. In the model considered in this paper the covariance matrix $R_{k}^{i}$ does not depend on *η* ^*i*^ and the Hessian becomes

$$H_{\eta }(l^{i}({\hat{\eta }}^{i}))\approx -\sum_{k=1}^{d_{i}}\nabla \varepsilon_{k}^{i}R_{k}^{i}{}^{{}^{-1}}\nabla \varepsilon_{k}^{i}-\Omega^{-1}.$$

In the first order conditional estimation (FOCE) method the mode of the individual log-likelihood function is used for ${\hat{\eta }}^{i}$, hence

$${\hat{\eta }}^{i}={\text{argmin}}_{\eta^{i}}\left\{-l^{i}(\eta^{i};Y|\theta )\right\},$$

in contrast to the first order (FO) method that uses ${\hat{\eta }}^{i}=0$.

For the STS model, the individual log-likelihood function in Eq (5) with the last three terms removed is maximized for each subject. In that case the uncertainty parameters *s* ^*i*^ and *S* ^*i*^ are estimated separately for each individual and *ϕ* ^*i*^ is set to 10. It should be noted that *k*_10,8_ is individual-specific for both models. To find the minima of the negative log-likelihood functions the Broyden–Fletcher–Goldfarb–Shanno (BFGS) optimization method is used (see [35]).

### Parameters

We distinguish between *model parameters* (e.g. transfer rates in the model) and *key parameters* (e.g. biological relevant parameters, often composed of several model parameters). The calculation of the key parameters VLDL_1_ secretion rate (SR), VLDL_2_ direct secretion rate (DSR), VLDL_2_ indirect secretion rate (IndSR) (i.e. the transfer from VLDL_1_ to VLDL_2_), VLDL_1_ and VLDL_2_ fractional clearance rate (FCR), VLDL_1_ fractional direct catabolic rate (FDCR), VLDL_1_ fractional transfer rate (FTR) is defined as

$$\begin{array}{l} \;{\text{VLDL}}_{1}\;{\text{SR}}^{i}=Q_{2}^{i}k_{L,2}^{i}d_{1}^{i},\\ \;{\text{VLDL}}_{1}\;{\text{FTR}}^{i}=\frac{Q_{6}^{i}k_{8,6}^{i}}{Q_{5}^{\text{i}}+Q_{6}^{\text{i}}+Q_{7}^{\text{i}}},\\ {\text{VLDL}}_{1}\;{\text{FDCR}}^{i}=\frac{Q_{6}^{i}k_{0,6}^{i}+Q_{7}^{i}k_{0,7}^{i}}{Q_{5}^{\text{i}}+Q_{6}^{\text{i}}+Q_{7}^{\text{i}}},\\ {\text{VLDL}}_{1}\;{\text{FDCR}}^{i}=\frac{Q_{6}^{i}k_{8,6}^{i}+Q_{6}^{i}k_{0,6}^{i}+Q_{7}^{i}k_{0,7}^{i}}{Q_{5}^{\text{i}}+Q_{6}^{\text{i}}+Q_{7}^{\text{i}}},\\ \;{\text{VLDL}}_{2}\;D{\text{SR}}^{i}=Q_{2}^{i}k_{L,2}^{i}(1-d_{1}^{i}),\\ {\text{VLDL}}_{2}\;{\text{IndSR}}^{i}=Q_{6}^{i}k_{8,6}^{i},\\ \;{\text{VLDL}}_{2}\;{\text{FCR}}^{i}=\frac{Q_{9}^{i}k_{0,9}^{i}+Q_{10}^{i}k_{10,7}^{i}}{Q_{8}^{\text{i}}+Q_{9}^{\text{i}}+Q_{10}^{\text{i}}}, \end{array}$$

The VLDL_1_ and VLDL_2_ pool sizes are defined as $Q_{V_{1}}^{i}$ and $Q_{V_{2}}^{i}$.

Although the mixed effects approach produces direct estimates of the distribution of model parameters, the key parameters are evaluated for each individual separately.

### Implementation

Both the mixed effects model and the STS model were implemented in MATLAB (The MathWorks Inc., Natick, MA).

### Statistical Analysis

Log-normally distributed variables are presented as geometric mean and coefficient of variation and were log-transformed before being compared using t-tests assuming equal variance. Mann Whitney test was used if the transformed data were not normally distributed. A p-value below 0.05 was considered significant. For power analyses a desired study power of 80% was used to calculate sample size.

## Supporting Information

S1 Fig Residual plot of enrichment data using reduced data. The residuals (model fit minus measurement data) for the three enrichment data sets (plasma leucine, VLDL_1_ and VLDL_2_) were plotted for the two methods (STS and NLME) and the two groups (Control and type 2 diabetes mellitus (DM2)). Both methods produced good fits to the data. Lines, mean of mixed effects approach (red) and STS approach (black); Areas, mean ± SD for mixed effects approach (red) and STS approach (black).
(DOCX)

S2 Fig Residual plot of enrichment data using truncated data. The residuals (model fit minus measurement data) for the three enrichment data sets (plasma leucine, VLDL_1_ and VLDL_2_) were plotted for the two methods (STS and NLME) and the two groups (Control and type 2 diabetes mellitus (DM2)). The NLME produced good fit to the data, even when extrapolating the curves between 4 and 8 hours. The STS approach in the other hand fails to produce a good fit for the extrapolated data. Lines, mean of mixed effects approach (red) and STS approach (black); Areas, mean ± SD for mixed effects approach (red) and STS approach (black).
(DOCX)

S1 File Data for 15 healthy individuals and 15 DM2 subjects. (CSV)

S2 File File format description for S1 File Data.csv file. (DOCX)

S1 Table Basic subject characteristics. Data from 15 healthy control subjects and 15 type 2 diabetes (DM2) patients were used in the study. As expected DM2 patients had higher plasma glucose and insulin concentrations and showed a typical dyslipidemia with elevated plasma triglycerides (TG), low high-density lipoprotein (HDL) cholesterol and smaller low-dense lipoprotein (LDL). Data is mean ± SD unless otherwise stated. ^a^ median (IQR); *, p<0.05 vs Control; **, p<0.01 vs Control; ***, p<0.001 vs Control.
(DOC)
