# Supplementary material for: Improved Estimation of Human Lipoprotein Kinetics with Mixed Effects Models
Source: PLoS One. 2015 Sep 30;10(9):e0138538. doi: 10.1371/journal.pone.0138538 (PMC4589417; doi:10.1371/journal.pone.0138538)
Supplement: S1 Fig — The residuals (model fit minus measurement data) for the three enrichment data sets (plasma leucine, VLDL1 and VLDL2) were plotted for the two methods (STS and NLME) and the two groups (Control and type 2 diabetes mellitus (DM2)). Both methods produced good fits to the data. Lines, mean of mixed effects approach (red) and STS approach (black); Areas, mean ± SD for mixed effects approach (red) and STS approach (black). (DOCX) [file pone.0138538.s001.docx]

**S1 Fig**
