# Supplementary material for: Improved Estimation of Human Lipoprotein Kinetics with Mixed Effects Models
Source: PLoS One. 2015 Sep 30;10(9):e0138538. doi: 10.1371/journal.pone.0138538 (PMC4589417; doi:10.1371/journal.pone.0138538)
Supplement: S2 Fig — The residuals (model fit minus measurement data) for the three enrichment data sets (plasma leucine, VLDL1 and VLDL2) were plotted for the two methods (STS and NLME) and the two groups (Control and type 2 diabetes mellitus (DM2)). The NLME produced good fit to the data, even when extrapolating the curves between 4 and 8 hours. The STS approach in the other hand fails to produce a good fit for the extrapolated data. Lines, mean of mixed effects approach (red) and STS approach (black); Areas, mean ± SD for mixed effects approach (red) and STS approach (black). (DOCX) [file pone.0138538.s002.docx]

**S2 Fig**
