# Supplementary material for: Improved Estimation of Human Lipoprotein Kinetics with Mixed Effects Models
Source: PLoS One. 2015 Sep 30;10(9):e0138538. doi: 10.1371/journal.pone.0138538 (PMC4589417; doi:10.1371/journal.pone.0138538)
Supplement: S2 File — (DOCX) [file pone.0138538.s004.docx]

**Data format**

The data in the file data.csv is formatted as follows:

ID;Time;Log Plasma leucine enrichment;Log VLDL1 enrichment;Log VLDL2 enrichment;VLDL1 pool size;VLDL2 pool size;Leucine injection;Diabetic

ID Subject ID (1-30)

Time Time point (hours)

Log Plasma leucine enrichment Logarithm of plasma leucine enrichment

Log VLDL1 enrichment Log of VLDL1 leucine (apoB) enrichment

Log VLDL2 enrichment Log of VLDL2 leucine (apoB) enrichment

VLDL1 pool size VLDL1 pool size

VLDL2 pool size VLDL2 pool size

Leucine injection Amount of leucine injected at t=0

Diabetic Control=0 or Diabetic=1
