# Supplementary material for: Improved Estimation of Human Lipoprotein Kinetics with Mixed Effects Models
Source: PLoS One. 2015 Sep 30;10(9):e0138538. doi: 10.1371/journal.pone.0138538 (PMC4589417; doi:10.1371/journal.pone.0138538)
Supplement: S1 Table — Data from 15 healthy control subjects and 15 type 2 diabetes (DM2) patients were used in the study. As expected DM2 patients had higher plasma glucose and insulin concentrations and showed a typical dyslipidemia with elevated plasma triglycerides (TG), low high-density lipoprotein (HDL) cholesterol and smaller low-dense lipoprotein (LDL). Data is mean ± SD unless otherwise stated. a median (IQR); *, p<0.05 vs Control; **, p<0.01 vs Control; ***, p<0.001 vs Control. (DOC) [file pone.0138538.s005.doc]

**S1 Table. Basic subject characteristics.**

|  | **Control (n=15)** | **DM2 (n=15)** |
| --- | --- | --- |
| **Age (years)** | 51 ± 7 | 55 ± 7 |
| **Weight (kg)** | 82.7 ± 8.3 | 89.5 ± 10.3 |
| **BMI (kg/m^2^)** | 26.4 ± 2.1 | 28.0 ± 3.9 |
| **Liver fat (%) ^a^** | 3.5 (2.00-5.00) | 11.0 (5.75-15)** |
| **Plasma TG (mmol/L) ^a^** | 1.42 (1.18-1.67) | 1.93 (1.52-2.51)* |
| **Cholesterol (mmol/L)** | 5.21 ± 0.94 | 4.83 ± 0.45 |
| **HDL cholesterol (mmol/L)** | 1.32 ± 0.22 | 1.01 ± 0.26** |
| **ApoB (mg/dl)** | 106 ± 20 | 119 ± 18 |
| **FFA (nmol/L)** | 558 ± 79 | 657 ± 174 |
| **Glucose (mmol/L)** | 5.8 ± 0.37 | 10.0 ± 2.96*** |
| **Insulin (U/L)** | 6.63 ± 2.75 | 11.28 ± 6.02* |

Data from 15 healthy control subjects and 15 type 2 diabetes (DM2) patients were used in the study. As expected DM2 patients had higher plasma glucose and insulin concentrations and showed a typical dyslipidemia with elevated plasma triglycerides (TG), low high-density lipoprotein (HDL) cholesterol and smaller low-dense lipoprotein (LDL). Data is mean ± SD unless otherwise stated. ^a^ median (IQR); *, p<0.05 vs Control; **, p<0.01 vs Control; ***, p<0.001 vs Control.
